# Striking a balance: Europe's green energy ambitions and the environmental impact of lithium

**DOI:** 10.1016/j.lanepe.2024.101090

**Published:** 2024-10-01

**Authors:** The Lancet Regional Health – Europe

As the world accelerates away from fossil fuels towards a green energy future powered by renewable and environmentally friendly sources, lithium has become essential in this transition. Lithium is a key component of high-capacity rechargeable batteries, which are used to store energy and power electric vehicles. Lithium's superior charge-to-weight ratio, high electrochemical potential, decreasing price and durability make it the preferred choice for electric vehicle batteries.

The increasing demand for electric vehicles in the European Union (EU) is set to drive a 60-fold surge in lithium demand by 2050. Recognising this, the EU has designated lithium as a critical raw material for transitioning to a low-carbon economy, with 60% of mined lithium now used for electric vehicle batteries. This supports the European Green Deal and the Fit for 55 package, targeting climate neutrality by 2050 and a 55% reduction in greenhouse gas emissions by 2030, respectively. While the global lithium supply is currently dominated by Australia, Chile, and China, the EU, under the Critical Raw Materials Act, aims to source at least 10% of its lithium domestically by 2030 to ensure a sustainable and resilient supply for its transition to green energy. As this pursuit intensifies, assessing and addressing the environmental impacts of lithium extraction becomes increasingly important.

Although lithium itself is not toxic, the extraction process results in considerable environmental risks, including air and water pollution, ecosystem disruption, and intensive water use—about 2 million litres of water is required per tonne of lithium when extracted from brines, leading to freshwater salinisation and substantial water loss. Additionally, hard rock mining emits 37 tonnes of CO_2_ per tonne of lithium produced. Considering these serious environmental risks, the challenge lies in balancing lithium's role in a sustainable green energy future with the need to protect the environment.

In Serbia, mass protests against a proposed lithium mine and allegations of exploratory drilling highlight fears of irreversible contamination of a major freshwater reservoir and fertile agricultural land, threatening biodiversity and food security. The absence of community involvement in negotiations and planning for the mines has further fuelled mistrust, particularly as the economic benefits from the mine are expected to be outweighed by those from agriculture. Similar resistance is seen in other middle-income countries, including Argentina and Bolivia, and in northern Portugal, where communities fear their land and livelihoods will be sacrificed for lithium extraction, deepening global inequalities.

Addressing the environmental impacts of lithium mining requires a multifaceted approach, from innovative recycling technologies to reducing reliance on lithium, funding research into mining alternatives, and stringent regulations on lithium mining.

Recycling or repurposing lithium from used batteries offers a partial solution, yet only about 5% of lithium-ion batteries are currently recycled. Lithium batteries in landfills cause environmental harm, including perfluoroalkyl and polyfluoroalkyl substance (PFAS) leakage. New European Commission regulations aim to boost recycling and reduce reliance on mined lithium. Although incineration of the batteries (after lithium removal) has been found to emit only minimal amounts of PFAS, and emerging extraction technologies show promise, widespread recycling and sustainable practices are still in development.

Shifting to smaller cars and thereby smaller batteries can also markedly reduce lithium demand and the strain on electricity grids when charging electric vehicles. In Norway, where electric vehicles account for 95% of new car sales—the highest figures in Europe—policies incentivise smaller, lighter cars. In the best-case scenario, data for the USA estimate that with smaller batteries, reduced car ownership, and widespread recycling, lithium demand could decline from 483,000 tonnes to just 40,000 tonnes by 2050—a 92% decrease.

While lithium is vital for the achievement of Europe's green energy goals, even with high electric vehicle adoption, a 2 °C rise in global temperatures remains likely. Therefore, reducing overall vehicle use, not just gas-powered vehicles, is crucial. Increasing the use of public transport, through initiatives like Luxembourg's free transit scheme, can reduce the need for electric vehicles and ease the strain on lithium resources. Prioritizing cycle lanes and creating pedestrian-friendly cities can further decrease reliance on personal vehicles and help alleviate the demand for lithium.

As lithium mining expands to support the green energy transition, it is crucial to recognise the environmental cost of this shift. Balancing progress with environmental protection will require robust regulations, adoption of sustainable practices, and active engagement with local communities. These measures are crucial to protect biodiversity and safeguard arable land. The EU could lead by setting stricter standards for lithium extraction, ensuring sustainable practices, and safeguarding local communities.

